# Diabetes Self-Management Smartphone Application for Adults With Type 1 Diabetes: Randomized Controlled Trial

**DOI:** 10.2196/jmir.2588

**Published:** 2013-11-13

**Authors:** Morwenna Kirwan, Corneel Vandelanotte, Andrew Fenning, Mitch J Duncan

**Affiliations:** ^1^Institute for Health and Social Science ResearchCentral Queensland UniversityNorth RockhamptonAustralia

**Keywords:** type 1 diabetes, mobile health, mobile phone, text message, education

## Abstract

**Background:**

Persistently poor glycemic control in adult type 1 diabetes patients is a common, complex, and serious problem initiating significant damage to the cardiovascular, renal, neural, and visual systems. Currently, there is a plethora of low-cost and free diabetes self-management smartphone applications available in online stores.

**Objective:**

The aim of this study was to examine the effectiveness of a freely available smartphone application combined with text-message feedback from a certified diabetes educator to improve glycemic control and other diabetes-related outcomes in adult patients with type 1 diabetes in a two-group randomized controlled trial.

**Methods:**

Patients were recruited through an online type 1 diabetes support group and letters mailed to adults with type 1 diabetes throughout Australia. In a 6-month intervention, followed by a three-month follow-up, patients (n=72) were randomized to usual care (control group) or usual care and the use of a smartphone application (Glucose Buddy) with weekly text-message feedback from a Certified Diabetes Educator (intervention group). All outcome measures were collected at baseline and every three months over the study period. Patients’ glycosylated hemoglobin levels (HbA1c) were measured with a blood test and diabetes-related self-efficacy, self-care activities, and quality of life were measured with online questionnaires.

**Results:**

The mean age of patients was 35.20 years (SD 10.43) (28 male, 44 female), 39% (28/72) were male, and patients had been diagnosed with type 1 diabetes for a mean of 18.94 years (SD 9.66). Of the initial 72 patients, 53 completed the study (25 intervention, 28 control group). The intervention group significantly improved glycemic control (HbA1c) from baseline (mean 9.08%, SD 1.18) to 9-month follow-up (mean 7.80%, SD 0.75), compared to the control group (baseline: mean 8.47%, SD 0.86, follow-up: mean 8.58%, SD 1.16). No significant change over time was found in either group in relation to self-efficacy, self-care activities, and quality of life.

**Conclusions:**

In adjunct to usual care, the use of a diabetes-related smartphone application combined with weekly text-message support from a health care professional can significantly improve glycemic control in adults with type 1 diabetes.

**Trial Registration:**

Australian New Zealand Clinical Trials Registry: ACTRN12612000132842; https://www.anzctr.org.au/Trial/Registration/TrialReview.aspx?ACTRN=12612000132842 (Archived by WebCite at http://www.webcitation.org/6Kl4jqn5u).

## Introduction

Persistently poor glycemic control in adult type 1 diabetes patients is a common, complex, and serious problem initiating significant damage to the cardiovascular, renal, neural, and visual systems [1]. In many patients, glycosylated hemoglobin levels (HbA_1c_) are unsatisfactory, with levels consistently above 8.0% [2]. In the pursuit of improving metabolic control, the importance of self-monitoring blood glucose is widely appreciated and recommended as a routine part of management in patients with type 1 diabetes [3]. There are a number of barriers to glycemic control in type 1 diabetes, including the fear of hypoglycemia and the demands of day-to-day management, in particular the need for frequent self-monitoring of blood glucose and regular adjustments in insulin dosing [4]. Additionally another difficulty is a patient’s logbook, either paper-based or electronic, that a clinician is presented with at a consultation. Clinicians often face a lack of information on which to base their advice regarding their patient’s self-care [5,6]. Utilizing mobile phone technology may help to overcome these difficulties.

The worldwide prevalence of mobile phones makes them a powerful platform for providing individualized health care delivered at the patient’s convenience. Several reviews have documented the effectiveness, potential, and challenges in using mobile phones to improve health outcomes in diabetes [7-14]. Growing evidence suggests that utilizing mobile phones may improve diabetes self-management and clinical outcomes; however, this evidence is much stronger for type 2 populations than type 1 populations [9].

In recent years, mobile phones have improved dramatically in both design and function, from simple call and text devices to the more sophisticated mini-personal computers known as smartphones. Smartphone owners are now more prevalent within the overall population than owners of traditional mobile phones [15]. Smartphones allow individual users to install, configure, and run specialized applications on their phone. Increasing numbers of people are using these applications to self-manage chronic diseases [13]. For example, Chomutare and colleagues [16] identified that in 2011 there were more than 260 diabetes-related iPhone applications available for download from the Apple online store.

A small number of prototypes of type 1 diabetes smartphone applications have been developed and tested in clinical settings [2,17-21]. However, with the plethora of low-cost and free self-management diabetes applications currently available in online markets (Apple iPhone, Google Android, BlackBerry, and Nokia Symbian), it is pertinent to examine their effectiveness when integrated in secondary care [16]. Therefore, the aim of this study was to examine the effectiveness of a freely available smartphone application combined with text-message feedback to improve glycemic control and other diabetes-related outcomes in adult patients with type 1 diabetes in a two-group randomized controlled trial.

## Methods

### Design

**Figure 1:**
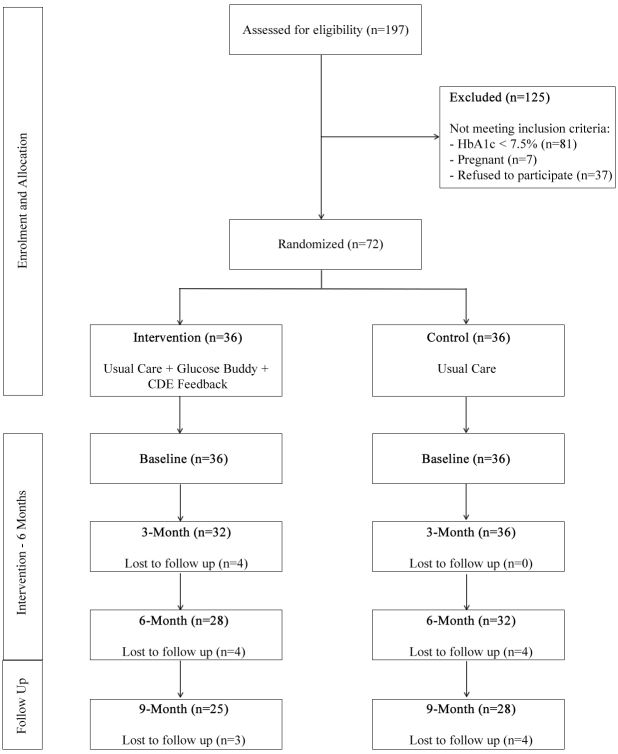
Participant flow. Note: Reason for subject “lost to follow-up” could not be determined as patients could not be re-contacted. CDE: Certified Diabetes Educator.

The study, utilizing a two-arm (usual care and intervention) randomized controlled trial with measures at baseline, 3, 6, and 9 months (Figure 1), was conducted with the assistance of a Certified Diabetes Educator (CDE). Participants were recruited nationally by means of an invitation letter sent to type 1 diabetes patients registered with Diabetes Australia in New South Wales (n=3809) and Queensland (n=3207), as well as an advertisement in a type 1 diabetes national newsletter (Yada Yada newsletter) emailed to more than 5000 recipients and promotion in an online community forum (Reality Check Forum). Study inclusion criteria were: (1) aged 18-65 years, (2) diagnosed with type 1 diabetes >6 months, (3) HbA_1c_ >7.5%, (4) treated with multiple daily injections or insulin pump, and (5) own a smartphone (iPhone). Patients were excluded if they were pregnant or already using a smartphone application to self-manage their diabetes. This study was approved by Central Queensland University Human Research Ethics Board.

After confirming eligibility (via phone call) and obtaining written informed consent (via email) from the patient and their primary diabetes health care practitioner (general practitioner or endocrinologist), the study coordinator randomized patients using a freely available online randomization program. A permuted block randomization design method was used during the 3-month rolling recruitment to ensure roughly equal numbers of patients were allocated to each comparison group [22]. There was no face-to-face contact between the patients and research team at any point in the study, which allowed participants to live anywhere in Australia.

### Intervention

**Figure 2:**
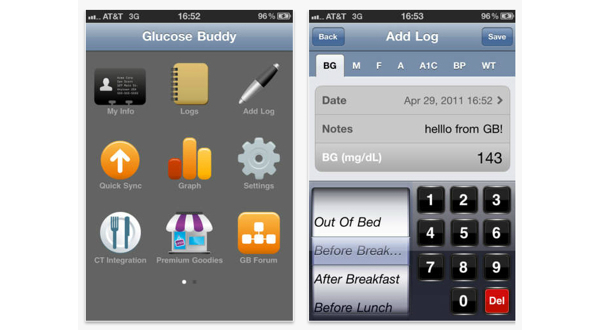
Glucose Buddy screenshots of the menu and adding a log.

**Figure 3:**
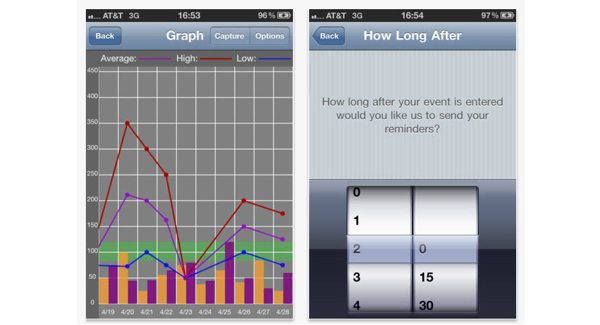
Glucose Buddy screenshots of the graphic display of logs and option to set up reminders.

Patients in both groups were asked to continue with their usual care, which included a visit to their primary diabetes health care practitioner every 3 months. Additionally, patients allocated to the intervention arm were given instructions to download the smartphone application named “Glucose Buddy”. Glucose Buddy is a freely available diabetes self-management iPhone application that allows users to manually enter blood glucose levels, insulin dosages, other medications, diet (food item in grams), and physical activities (minutes) [16,23]. Users can also view their data on a customizable graph and export this information via email (Figures 2 and 3). Glucose Buddy was developed by SkyHealth LLC and was first made available on iTunes (Apple online store) in October 2008. Glucose Buddy has been reported to be the most downloaded diabetes management software on iOS, with downloads in excess of 100,000. There was no minimum amount of logging required and intervention patients were able to utilize the accompanying Glucose Buddy website to log diabetes parameters at their discretion.

The information logged in the Glucose Buddy application was reviewed by a CDE via a Web interface on a weekly basis. All patients in the intervention arm were sent a minimum of 1 personalized text-message communication per week for the first 6 months of the study. At the 6-month timeframe, all text-message communication ceased.

### Measures

All measures were collected at baseline and every 3 months over the 9-month study period for both groups (making 4 time points in total). The primary outcome measure was change in glycemic control assessed by HbA_1c_, which was collected by a pathology lab at the request of the patients’ general practitioner or endocrinologist as per usual care (every 3 months) and then forwarded to the research team. The secondary outcome measures, being diabetes-related self-efficacy, self-care activities, and quality of life, were collected via a Web-based survey. Details to access this survey were emailed to patients.

Diabetes-related self-efficacy was measured using the short form of the Diabetes Empowerment questionnaire (DES-SF). The DES-SF questionnaire measures eight conceptual dimensions relevant to the management of diabetes: (1) assessing the need for change, (2) developing a plan, (3) overcoming barriers, (4) asking for support, (5) supporting oneself, (6) coping with emotion, (7) motivating oneself, and (8) making diabetes care choices appropriate for one’s priorities and circumstances. Patients respond to all items on a 5-point Likert scale, with 1-strongly disagree to 5-strongly agree. Higher scores on DES-SF reflect a better self-efficacy. Research has shown that DES-SF is a valid and reliable measure of overall diabetes-related psychosocial self-efficacy [24,25].

The Summary of Diabetes Self-Care Activities (SDSCA) measure was used for assessing diabetes self-care behaviors. The SDSCA contains 6 scales: (1) general dietary behavior, (2) specific dietary behavior, (3) glucose monitoring, (4) exercise, (5) foot care, and (6) smoking. Only the first four listed scales were used in this study. Higher scores on the SDSCA reflect a greater number of days per week that self-care activities are carried out (range 0-7). Psychometric analyses support the construct validity and internal consistency reliability of SDSCA in an adult diabetes population [26].

The valid and reliable Diabetes Quality of Life (DQOL) scale [27] was used to assess the three aspects of quality of life directly related to diabetes: diabetes satisfaction, impact, and worry. All the subscales were included. Patients respond to all items on a 5-point Likert scale. A score of 1 indicates “always affected”, “always worried”, or “never satisfied”. A score of 5 represents “no impact”, “no worries”, or “always satisfied”. Higher scores on DQOL scales reflect better quality of life.

Intervention participants’ engagement with the intervention was also measured in terms of text-message communications with the CDE and the utilization of the Glucose Buddy application. Specifically, the number of text messages sent to patients and the number of text-message responses were collected as well as the number of logs (blood glucose, insulin, physical activity, and diet) entered by patients in the Glucose Buddy application.

### Statistical Analysis

Demographic characteristics of participants and baseline data for all measures were compared between both study groups to detect differences at baseline using a series of independent sample *t* tests and chi-square tests. Logistic regression analyses were conducted to evaluate whether participant characteristics (age [years], duration of diabetes [months/years], gender, insulin pump use [Y/N], and baseline HbA_1c_) were related to dropout (completed vs didn’t complete all assessments) during the study. This statistical method is common when evaluating the characteristics that may be related to attrition examined as a dichotomous outcome. Primary (HbA_1c_) and secondary outcomes (diabetes-related self-efficacy, quality of life, and self-care) measures were analyzed using linear mixed effects models for repeated measures. Linear mixed model analysis allows for inclusion of cases with missing data, without replacement of missing values, and therefore includes all randomized patients. The linear mixed model analysis used group and the covariates of age (years), gender, and diabetes duration (years) as fixed effects. The “Type III Wald test” was used to test overall statistical significance of the effects. Linear regression analysis was conducted to analyze whether engagement in the study by the intervention group was predictive of change in HbA_1c_; it allows to assess whether patients who engaged more in the intervention, in terms of text-message communications and logging parameters in the Glucose Buddy application, had a greater change in HbA_1c_. Statistical significance was defined as *P*<.05 for all analysis and conducted using SPSS for Windows (Version 18.0).

The sample size was calculated on the expected difference in mean (1.5%) in the primary outcome variable (HbA_1c_), and the logistically maximum available sample size was 36 patients per group based on part-time work status of the CDE. We allowed for a dropout of 11% (4 per group), consistent with dropout rates reported in recent reviews of similar studies [9,10], and variation in baseline (HbA_1c_=1.80) similar to previous studies [28]. Based on these parameters and using an alpha of .05 and 90% power, the estimated sample size was 68 in total and subsequently increased to 72 in line with the maximum caseload of the CDE [29].

## Results

### Participant Characteristics

In total, 197 adults with type 1 diabetes registered their interest online or via phone call to the research team and were assessed for eligibility (Figure 1), with 125 excluded for not meeting the inclusion criteria. Seventy-two individuals were randomized to the two groups. Linear mixed model analysis allows for inclusion of cases with missing data, without replacement or imputation of missing values. Therefore, this analysis approach includes all available data of randomized patients at each time point as indicated in Figure 1. Table 1 provides an outline of the participant’s characteristics. Mean age of patients was 35.20 years (SD 10.43), 39% (28/72) were male, and patients had been diagnosed with type 1 diabetes for a mean of 18.94 years (SD 9.66). In total, 38% (27/72) of patients were using an insulin pump, with no significant difference between groups, χ^2^_1_=0.59, *P*=.81. The intervention group had a significantly higher (*P*=.02) baseline HbA_1c_ (mean 9.08, SD 1.18) than the control group (mean 8.47, SD 0.86) and reported a healthier diet (mean 3.56, SD 1.70 healthy days per week for the intervention group versus mean 2.60, SD 1.98 days for the control group, *P*=.03). There were significantly (*P*=.02) more females (75%, 27/36) in the control group. No other baseline differences were observed between groups. Dropout was 26% (11 males, 8 females, 19/72) with logistic regression analysis revealing no significant difference in age, gender, diabetes duration, insulin pump use, and baseline HbA_1c_ among those that completed the study and those that were lost to follow up.

**Table 1:** Participant characteristics at baseline.

		Overall, n=72	Control, n=36	Intervention, n=36	*P* value
Gender (M/F), n		28/44	9/27	19/17	.02
Age (years), mean (SD)		35.20 (10.43)	34.42 (10.26)	35.97 (10.67)	.51
Diabetes duration (years), mean (SD)		18.94 (9.66)	18.19 (9.77)	19.69 (9.64)	.53
Insulin pump use, n		27/72	13/36	14/36	.81
**HbA**_**1c**_ **, mean (SD)**					
	Total group	8.78 (1.07)	8.47 (0.86)	9.08 (1.18)	.02
	Male	8.79 (1.31)	8.17 (0.65)	9.10 (1.45)	.08
	Female	8.77 (0.90)	8.57 (0.91)	9.07 (0.84)	.08

### Intervention Effects

As outlined in Table 2, the linear mixed model revealed that there was a significant interaction effect between groups for HbA_1c_ (*F*_1,246_=20.07, *P*<.001)*.* The intervention group had a significant decrease in HbA_1c_ (mean −1.10, SD 0.74, *P*<.001) over the 9-month study, compared to the control group which had a non-significant increase (mean 0.07, SD 0.99). There was a statistically significant change in the diabetes self-care measure of specific diet over time, but there was no difference between the two groups. No significant differences were observed for all other outcomes.

### Engagement by Intervention Patients

Intervention patients’ engagement with the Glucose Buddy application, in terms of the number of logs and text messages communicated between patients and the CDE, is outlined in Table 3. Over the 6-month intervention period, the CDE sent 1714 text messages in total, which equates to approximately 2 text messages per patient per week. Patients sent in total 559 text messages to the CDE over the 6-month period. The first month of the study was used for the CDE and the intervention group patients to establish a relationship—they never met in person. Thus, the text messages sent to patients (mean 9.75, SD 1.96) in the first month and those received by the CDE (mean 6.47, SD 3.92) are higher than the average of the other five months. Using the Glucose Buddy application, patients logged 24,720 diabetes parameters in total: 54.00% (13,349/24,720) of the logs related to blood glucose levels, 33.00% (8158/24,720) to insulin, 12.00% (2966/24,720) to diet, and 1.00% (247/24,720) to exercise. Linear regression analysis revealed no significant relationship between level of engagement and change in HbA_1c_ in the intervention, as measured by text messages sent to the patients, text messages received by the CDE, and the number of logs entered in the Glucose Buddy application.

### Text Message Themes

The content of the text messages sent to and received from patients fell into four broad thematic categories covering feedback on logs, diabetes questions, educational tips, and positive reinforcement. Examples of text messages for each category are outlined in Table 4.

### Costs Incurred

The Glucose Buddy application was freely available. A text-messaging software program was used to text message patients. Total cost over the study was $290.93 AUD; this equates to $8.08 per patient (n=36). The CDE spent on average 3 hours per week reviewing patients’ logs and text messaging patients in the intervention group. This equated to 5 minutes per patient (n=36) per week (72 hours in total over 6-month period). A CDE hourly rate is approximately $28.85; thus, the total cost over the study was $2077.20 AUD.

**Table 2:** Results of linear mixed model analysis for diabetes glycemic control, self-efficacy, self-care, and quality of life measures.

			Overall^a^	Control^b^	Intervention^c^	Overall *F* statistic, (*df*=1,246)		
			Mean (SD)	Mean (SD)	Mean (SD)	Time	Group	Time x Group
**HbA**_**1c**_ **(%)**								
		Baseline	8.78 (1.07)	8.47 (0.86)	9.08 (1.18)^d^			
		3-month	8.27 (0.86)	8.23 (0.89)	8.32 (0.84)	28.79^e^	10.55^d^	20.07^e^
		6-month	8.22 (0.91)	8.43 (1.00)	7.97 (0.73)			
		9-month	8.21 (1.05)	8.58 (1.16)	7.80 (0.75)			
**SDSCA** ^f^								
	**General Diet**							
		Baseline	3.81 (2.06)	4.19 (1.88)	3.42 (2.19)			
		3-month	4.10 (2.00)	3.97 (2.13)	4.23 (1.86)	5.30^d^	1.92	3.40
		6-month	4.36 (1.86)	4.16 (1.98)	4.59 (1.73)			
		9-month	4.37 (1.83)	4.14 (1.85)	4.62 (1.80)			
	**Specific Diet**							
		Baseline	3.08 (1.89)	2.60 (1.98)	3.56 (1.70) ^d^			
		3-month	3.10 (1.63)	3.00 (1.76)	3.22 (1.48)	0.52	6.90^d^	3.36
		6-month	3.68 (1.63)	3.56 (1.66)	3.80 (1.60)			
		9-month	3.93 (1.61)	4.05 (1.43)	3.80 (1.82)			
	**Exercise**							
		Baseline	2.74 (2.09)	2.92 (2.12)	2.57 (2.08)			
		3-month	2.45 (2.12)	2.53 (2.31)	2.36 (1.91)	1.44	2.22	0.93
		6-month	2.83 (1.95)	3.06 (1.97)	2.55 (1.92)			
		9-month	2.96 (1.79)	2.82 (1.75)	3.12 (1.86)			
	**Glucose Testing**							
		Baseline	5.46 (2.04)	5.51 (2.08)	5.40 (2.03)			
		3-month	5.88 (1.64)	5.75 (1.65)	6.02 (1.64)	3.65	1.42	1.86
		6-month	6.10 (1.56)	6.02 (1.67)	6.20 (1.46)			
		9-month	5.92 (1.62)	5.61 (1.95)	6.28 (1.06)			
**DES-SF** ^g^								
		Baseline	3.62 (0.77)	3.62 (0.65)	3.62 (0.89)			
		3-month	3.78 (0.64)	3.70 (0.65)	3.88 (0.61)	0.01	0.16	0.001
		6-month	3.73 (0.77)	3.64 (0.81)	3.82 (0.73)			
		9-month	3.61 (0.72)	3.62 (0.74)	3.60 (0.71)			
**DQOL** ^h^								
	**Satisfaction**							
		Baseline	3.14 (0.61)	3.09 (0.55)	3.20 (0.66)			
		3-month	3.22 (0.61)	3.11 (0.54)	3.35 (0.67)	0.55	1.42	0.01
		6-month	3.32 (0.60)	3.23 (0.62)	3.43 (0.58)			
		9-month	3.35 (0.66)	3.29 (0.65)	3.42 (0.68)			
	**Impact**							
		Baseline	3.70 (0.53)	3.66 (0.54)	3.75 (0.52)			
		3-month	3.81 (0.57)	3.75 (0.56)	3.89 (0.58)	0.58	0.71	0.07
		6-month	3.83 (0.61)	3.74 (0.61)	3.94 (0.62)			
		9-month	3.85 (0.53)	3.77 (0.55)	3.93 (0.52)			
	**Worry**							
		Baseline	3.98 (0.66)	3.90 (0.78)	4.06 (0.52)			
		3-month	4.10 (0.70)	4.01 (0.77)	4.19 (0.61)	1.77	1.26	0.70
		6-month	4.16 (0.70)	3.99 (0.82)	4.35 (0.46)			
		9-month	4.15 (0.63)	3.99 (0.76)	4.34 (0.36)			

^a^Number of participants included in both groups at each time point is: baseline n=72, 3 month n=68, 6 month n=60, 9 month n=53.

^b^Number of participants included in the Intervention group at each time point is: baseline n=36, 3 month n=32, 6 month n=28, 9 month n=25.

^c^Number of participants included in the Control group at each time point is: baseline n=36, 3 month n=36, 6 month n=32, 9 month n=28.

^d^ *P*<.05

^e^ *P*<.001

^f^SDSCA: Summary of Diabetes Self Care Activities

^g^DES-SF: Diabetes Empowerment Scale

^h^DQOL: Diabetes Quality of Life

**Table 3:** Engagement with intervention.

	Month 1	Month 2	Month 3	Month 4	Month 5	Month 6
Average number of text messages sent to patients, mean (SD)	9.75 (1.96)	6.67 (1.47)	7.58 (2.12)	7.11 (1.91)	8.56 (2.26)	7.94 (2.52)
Median number of text messages sent to patients	9	7	7	7.5	8	8
Total number of text messages sent to patients	351	240	273	256	308	286
Average number of text messages received by CDE^a^ , mean (SD)	6.47 (3.92)	2.36 (2.82)	2.03 (2.52)	1.11 (1.30)	2.22 (2.84)	1.33 (2.46)
Median number of text messages received by CDE	6	2	1	1	1.5	0
Total number of text messages received by CDE	233	85	73	40	80	48
Glucose Buddy Logs, mean (SD)	187.53 (137.41)	137.22 (143.39)	92.03 (109.66)	96.02 (129.06)	89.03 (107.14)	84.83 (153.47)
Glucose Buddy Logs, total N	6751	4940	3313	3457	3205	3054

^a^CDE: Certified Diabetes Educator

**Table 4:** Text messages.

Themes	Examples
Feedback on logs (blood glucose, insulin, diet)	*Hi <patient name>- Another pretty good week - just a bit concerned about some odd higher levels in the morning - looks like some of these are forgotten doses - would that be right? Otherwise all are getting better and no real hypos. Be aware you may need to tweak basal if those highs are not related to forgotten doses. Your thoughts?* [Sent from Certified Diabetes Educator] * Hi Veronica- Yes I have been having my breakfast later after I arrive to work, I get distracted and then can’t remember if I have had my insulin or not. I think I will just have a piece of fruit and insulin before I leave for work to prevent the high. I think the basal is ok but I will check over next couple of days, thanks for your help.* [Response from Patient]
Diabetes questions	*Hi <patient name>- I’ve been thinking about your CHO ratio. Have you ever done a basal check - which is simply to just have basal insulin overnight & on waking up and having no CHO & omit fast acting insulin for breakfast or lunch & just check how your levels go? If basal is right you should stay pretty constant. It’s a good place to start - if basal is right we can start on CHO ratio with novo?* [Sent from Certified Diabetes Educator] *Hi Veronica, I have never heard of a basal check but I did this, as you said and you can see on my download I was 12 -2 hours after tea (before bed) and went down to 8 when I woke up. So I now understand, as you say I need to adjust my CHO ratio with the evening meals so my after tea BSL is lower. Will adjust and let you have a look at levels next week.* [Response from Patient]
Educational tips	*Hi <patient name>- Great logging this week - just a few Xmas tips - don’t forget to give extra fasting acting insulin for extra treats - remember extra alcohol will make you low so eat carbs if you drink! Today I will be standing by to answer Xmas queries so please feel free to send them through. Merry Xmas!* [Sent from Certified Diabetes Educator] *I didn’t realize that alcohol made my BSL lower. That explains a lot - I have had some bad hypos in the morning after a big night out. I usually give myself extra insulin for each beer I drink. Should I give any fast acting insulin for alcohol at all or just eat extra CHO serves?* [Response from Patient]
Positive reinforcement	*Hi <patient name>- Great logging this week –WOW I’m thrilled with your HbA*_*1c*_ *& your positive comment about it being the best in 6 years. It is a testament to you because you are keeping such good track on your own adjustments and control. Looks like another great week again!* [Sent from Certified Diabetes Educator] *Thanks, after I was first diagnosed, I kept a log but then I didn’t bother to keep track of anything because I just always felt out of control. By getting direct feedback, I have understood more about the finer details about insulin/carbs/exercise and alcohol which has made me less reactive and think more about my adjustments.* [Response from Patient]

## Discussion

### Principal Findings

In adjunct with usual care, use of the Glucose Buddy application combined with weekly text-message feedback from a CDE led to a significant decrease in HbA_1c_ in comparison to a control group receiving only usual care. While regression to the mean cannot be ruled out, these results suggest that the intervention was effective. Improvements in HbA_1c_ of this magnitude in type 1 diabetes patients have been found previously in a mobile phone study [2] but are rare [17,19,30,31]. All patients in our study had poorly controlled diabetes at baseline; however, the intervention group had a significantly higher HbA_1c_ at baseline (mean 9.08%, SD 1.18 vs mean 8.47%, SD 0.86) and thus had a greater potential to improve their glycemic control. However, a meta-analysis of mobile intervention studies on diabetes glycemic control demonstrated only a 0.3% improvement for type 1 patients [9]; this demonstrates the success of the current intervention (a decrease of 1.1% in the intervention group) despite the baseline differences observed between groups.

It is unclear what mediated the change in HbA_1c_ in the current study as our analysis did not show a significant association between Glucose Buddy application usage, text message communications, and change in HbA_1c_. Those patients who used the Glucose Buddy application and text messaged the CDE most frequently did not have a greater change in HbA_1c_ than those who used them less. This is supported in previous research which has found no association between engagement (increased contact between patient and clinician) and clinical improvement [32-36]. Perhaps the improvement in glycemic control in our study may be attributed to offering both a smartphone application and a website to log parameters. Mulvaney and colleagues [10] identified in their systematic review that diabetes studies which included a mobile phone and Internet component showed a greater reduction in HbA_1c_ (0.7% vs 0.4%) when compared to studies with only a mobile phone component. Unfortunately, we do not know (due to software constraints) the extent to which each component was used by patients during our study. Alternatively, it may be that the user engagement/health status relationship in IT-based interventions is more complex than measured in the current study. The estimated sample size in the current study was based on logistical constraints and the ability to detect a change in the primary outcome; as such, the study may have been underpowered for the mediation analysis between platform usage and change in the primary outcome.

The current study did not find an improvement in self-efficacy in the intervention group. Previous Web and mobile phone diabetes studies have found positive changes in self-efficacy [33,37-39]; however, this change in self-efficacy is not always correlated with a change in HbA_1c_ [38,39]. Perhaps the relation between self-efficacy and HbA_1c_ is less important than previously assumed [24]. There was no change in quality of life in our study within and between groups over time. This finding is supported by previous telemedicine studies in type 1 diabetes that were also unable to observe an improvement in quality of life despite improvements in HbA_1c_ [2,32,40-42]. This may be due to the fact that diabetes self-management remains a burden despite short-term improvements in glycemic control and effects on quality of life might only manifest themselves in the longer term [43]. Additionally, there was no change in self-care activities for either group over time. This was unexpected considering that our intervention group had a significant improvement in HbA_1c_, which is traditionally correlated with an increase in frequency in blood-glucose testing [8,38,44,45].

A recent systematic review highlighted that the level of engagement of participants in mobile intervention diabetes studies is underreported [10]. Our intervention group (n=36) logged 24,720 parameters in the Glucose Buddy application over a 6-month period of time. This is comparative to Farmer and colleagues [35], whose intervention group (n=47) logged 29,795 blood glucose results over a 9-month period of time. Patients in our study limited their self-monitoring practices to those indicators with high importance to the self-management of their condition. For example, blood glucose was logged 54.00% of the time, whereas exercise was logged only 1.00% of time. Our intervention patients sent 559 text messages in total, equating to a mean of 15.5 (559/36) per participant over the entire intervention period. Similarly in a mobile phone-based type 1 diabetes study, intervention patients sent a total of 1180 text messages, a mean of 18.4 text messages per person [34]. The CDE in our study spent approximately 5 minutes per week per patient to monitor and provide feedback. This is comparative to time taken by clinical practitioners in Benhamou and Melki’s [19] research who spent on average 4.5 minutes per week per patient.

Despite there being an overall high level of engagement by patients in this intervention, this did decrease over the study period. Keeping patients adherent to treatment and maintaining engagement over time are significant problems that have been documented in health care [46]. This is also true of behavioral interventions across varied intervention delivery modalities, especially the Internet [47-49]. Issues in patient engagement and frequency of contacts have also been noted in mobile research [11,50]. This may be attributed to actual decreases in self-management activities or may be due to individuals becoming more self-aware of their daily behaviors/ health status due to self-monitoring and education from CDE and as such becoming less reliant on the need to self-monitor via intervention platforms. This potential effect requires further examination.

Intervention patients in our study were not provided with any education or training on how to use the Glucose Buddy smartphone application or website. Previous diabetes telemedicine studies have documented education and training sessions provided to intervention patients on the use of the technology under investigation. These training sessions ranged from 15 minutes [51], 1 hour [39,44,52], 6 hours [53], and even 1 day [38,54]. Given the high usage of the application in combination with providing no training on how to use it, it is likely that the design and usability of the application were not barriers to usage. Research has shown that to be competitive and encourage uptake and long-term adoption, smartphone applications should be intuitive and user-friendly [55-60]. This is especially relevant when investigating the effectiveness of smartphone applications to improve self-care in type 1 diabetes patients; it is a disease with no endpoint, requiring long-term self-management that can only be facilitated by user-friendly applications.

There is much enthusiasm from researchers concerning real-time feedback using mobile technology to assist patients with diabetes self-management [2,61-63]. It has been espoused that the future role of smartphones in diabetes care relates to providing patients with sophisticated applications that automatically upload blood glucose levels from glucometers and provides systematic advice concerning insulin dosage—perhaps sending this information wirelessly to an insulin pump. Indeed, smartphone applications hold great potential for taking diabetes self-management to a new level. We do not dispute that automation of the process may decrease the burden on patients compared to manual entry. However, we would argue that there is still value in manual entry of glucose levels, insulin, diet, and physical activity, despite being onerous. Problem solving is a core component of diabetes self-management [64] and, if all facets of measurement are automated, this may actually result in less awareness and thus less reflection by the patient on their condition; this might paradoxically lead to poorer self-management and negative clinical outcomes in the long term, with the added risk that a failure of the automated technology (eg, empty battery) might lead to panic and wrong decisions taken by patients. Additionally, the development of decision-support systems [65], although designed using medical information and clinical guidelines, is focused on reducing the practitioner element in the feedback process. As outlined in a recent review on mobile intervention design in diabetes [10], those studies that had the greatest impact on HbA_1c_ made use of clinician involvement and feedback. The importance of the human element should not be discounted.

### Limitations

There are limitations to our study that should be noted. First, this study was a randomized controlled trial with a small sample conducted over a short duration. Due to the dropout of patients, the study may not have been powered sufficiently to detect differences between groups for the secondary outcome measures. Second, there were differences in glycemic control and gender between groups at baseline. Third, although patients in the control group were instructed not to use any mobile applications to self-manage their diabetes during the study period, it is possible they did.

### Conclusions

Despite these limitations, we did find that integrating a smartphone application into secondary care was effective in improving glycemic control in patients with type 1 diabetes. Our findings can be applied to adults with poorly controlled type 1 diabetes that own a smartphone, though larger studies over a longer duration need to be conducted to validate our findings.

## Multimedia Appendix 1

CONSORT-EHEALTH checklist V1.6.2 [66].

## Abbreviations

CDE: Certified Diabetes Educator
DES-SF: Diabetes Empowerment Scale
DQOL: Diabetes Quality of Life
HbA1c: glycosylated hemoglobin
SDSCA: Summary of Diabetes Self Care Activities
