# Supplementary material for: Diabetes Self-Management Smartphone Application for Adults With Type 1 Diabetes: Randomized Controlled Trial
Source: J Med Internet Res. 2013 Nov 13;15(11):e235. doi: 10.2196/jmir.2588 (PMC3841374; doi:10.2196/jmir.2588)
Supplement: Supplementary file 1 [file jmir_v15i11e235_app1.pdf]

CONSORT-EHEALTH Checklist V1.6.2 Report

(based on CONSORT-EHEALTH V1.6), available at [http://tinyurl.com/consort-ehealth-v1-6].

2588

Date completed

10/30/2013 2:21:40

by

Morwenna Kirwan

Randomized Controlled Trial of a Diabetes Self-Management Smartphone Application for Adults with Type 1 Diabetes

TITLE

1a-i) Identify the mode of delivery in the title

Randomized Controlled Trial of a Diabetes Self-Management Smartphone Application for Adults with Type 1 Diabetes

1a-ii) Non-web-based components or important co-interventions in title

This intervention was delivered via smartphone.

1a-iii) Primary condition or target group in the title

Randomized Controlled Trial of a Diabetes Self-Management Smartphone Application for "Adults with Type 1 Diabetes"

ABSTRACT

1b-i) Key features/functionalities/components of the intervention and comparator in the METHODS section of the ABSTRACT

The aim of this study was to examine the effectiveness of a freely available smartphone application combined with text-message feedback from a certified diabetes educator to improve glycemic control and other diabetes-related outcomes in adult patients with type 1 diabetes in a two-group randomized controlled trial.

Patients were recruited through an online type 1 diabetes support group and letters mailed to adults with type 1 diabetes throughout Australia. In a six-month intervention, followed by a three-month follow-up, patients (n=72) were randomized to usual care (control group) or usual care and the use of a smartphone application (Glucose Buddy) with weekly text-message feedback from a certified diabetes educator (intervention group). All outcome measures were collected at baseline and every three months over the study period. Patients' glycosylated hemoglobin levels (HbA1c) were measured with a blood test and diabetes-related self-efficacy, self-care activities, and quality of life were measured with online questionnaires.

1b-ii) Level of human involvement in the METHODS section of the ABSTRACT

In a six-month intervention, followed by a three-month follow-up, patients (n=72) were randomized to usual care (control group) or usual care and the use of a smartphone application (Glucose Buddy) with weekly text-message feedback from a certified diabetes educator (intervention group). All outcome measures were collected at baseline and every three months over the study period. Patients' glycosylated hemoglobin levels (HbA1c) were measured with a blood test and diabetes-related self-efficacy, self-care activities, and quality of life were measured with online questionnaires.

1b-iii) Open vs. closed, web-based (self-assessment) vs. face-to-face assessments in the METHODS section of the ABSTRACT

In a six-month intervention, followed by a three-month follow-up, patients (n=72) were randomized to usual care (control group) or usual care and the use of a smartphone application (Glucose Buddy) with weekly text-message feedback from a certified diabetes educator (intervention group). All outcome measures were collected at baseline and every three months over the study period. Patients' glycosylated hemoglobin levels (HbA1c) were measured with a blood test and diabetes-related self-efficacy, self-care activities, and quality of life were measured with online questionnaires.

1b-iv) RESULTS section in abstract must contain use data

Results: The mean age of patients was 35.20±10.43 years (28 male /44 female), 39% (28/72) were male, and patients had been diagnosed with type 1 diabetes for a mean of 18.94±9.66 years. Of the initial 72 patients, 53 completed the study (25 intervention, 28 control group). The intervention group significantly improved glycemic control (HbA1c) from baseline (9.08±1.18%) to nine-month follow-up (7.80±0.75%), compared to the control group (baseline 8.47±0.86%, follow-up 8.58±1.16%). No significant change over time was found in either group in relation to self-efficacy, self-care activities, and quality of life.

1b-v) CONCLUSIONS/DISCUSSION in abstract for negative trials

Primary outcome did change.

INTRODUCTION

2a-i) Problem and the type of system/solution

Persistently poor glycemic control in adult type 1 diabetes patients is a common, complex, and serious problem initiating significant damage to the cardiovascular, renal, neural, and visual systems [1]. In many patients, glycosylated hemoglobin levels (HbA1c) are unsatisfactory, with levels consistently above 8.0% [2]. In the pursuit of improving metabolic control, the importance of self-monitoring blood glucose is widely appreciated and recommended as a routine part of management in patients with type 1 diabetes [3]. There are a number of barriers to glycemic control in type 1 diabetes, including the fear of hypoglycemia and the demands of day-to-day management, in particular the need for frequent self-monitoring of blood glucose and regular adjustments in insulin dosing [4]. Additionally another difficulty is a patient's logbook, either paper-based or electronic, that a clinician is presented with at a consultation. Clinicians often face a lack of information on which to base their advice regarding their patient's self-care [5,6]. Utilizing mobile phone technology may help to overcome these difficulties.

2a-ii) Scientific background, rationale: What is known about the (type of) system

A small number of prototypes of type 1 diabetes smartphone applications have been developed and tested in clinical settings [2,17-21]. However, with the plethora of low-cost and free self-management diabetes applications currently available in online markets (Apple iPhone, Google Android, BlackBerry, and Nokia Symbian), it is pertinent to examine their effectiveness when integrated in secondary care [16].

METHODS

3a) CONSORT: Description of trial design (such as parallel, factorial) including allocation ratio

Therefore, the aim of this study was to examine the effectiveness of a freely available smartphone application combined with text-message feedback to improve glycemic control and other diabetes-related outcomes in adult patients with type 1 diabetes in a two-group randomized controlled trial.

3b) CONSORT: Important changes to methods after trial commencement (such as eligibility criteria), with reasons

There were no changes to eligibility criteria after trial commencement.

3b-i) Bug fixes, Downtimes, Content Changes

There were no issues with bugs, no downtime and no content changes.

4a) CONSORT: Eligibility criteria for participants

Study inclusion criteria were: (1) aged 18-65 years, (2) diagnosed with type 1 diabetes >6 months, (3) HbA1c >7.5%, (4) treated with multiple daily injections or insulin pump, and (5) own a smartphone (iPhone). Patients were excluded if they were pregnant or already using a smartphone application to self-manage their diabetes. This study was approved by Central Queensland University Human Research Ethics Board.

4a-i) Computer / Internet literacy

It was part of the eligibility criteria that participants own a smartphone. It was assumed if they owned a smartphone - they could use it.

4a-ii) Open vs. closed, web-based vs. face-to-face assessments:

Participants were recruited nationally by means of an invitation letter sent to type 1 diabetes patients registered with Diabetes Australia in New South Wales (n=3809) and Queensland (n=3207), as well as an advertisement in a type 1 diabetes national newsletter (Yada Yada newsletter) emailed to more than 5000 recipients and promotion in an online community forum (Reality Check Forum).

4a-iii) Information giving during recruitment

After confirming eligibility (via phone call) and obtaining written informed consent (via email) from the patient and their primary diabetes health care practitioner (general practitioner or endocrinologist), the study coordinator randomized patients using a freely available online randomization program. A permuted block randomization design method was used during the 3-month rolling recruitment to ensure roughly equal numbers of patients were allocated to each comparison group [22]. There was no face-to-face contact between the patients and research team at any point in the study, which allowed participants to live anywhere in Australia.

4b) CONSORT: Settings and locations where the data were collected

There was no face-to-face contact between the patients and research team at any point in the study, which allowed participants to live anywhere in Australia.

The primary outcome measure was change in glycemic control assessed by HbA1c, which was collected by a pathology lab at the request of the patients' general practitioner or endocrinologist as per usual care (every 3 months) and then forwarded to the research team. The secondary outcome measures, being diabetes-related self-efficacy, self-care activities, and quality of life, were collected via a Web-based survey. Details to access this survey were emailed to patients.

4b-i) Report if outcomes were (self-)assessed through online questionnaires

There was no face-to-face contact between the patients and research team at any point in the study, which allowed participants to live anywhere in Australia.

The primary outcome measure was change in glycemic control assessed by HbA1c, which was collected by a pathology lab at the request of the patients' general practitioner or endocrinologist as per usual care (every 3 months) and then forwarded to the research team. The secondary outcome measures, being diabetes-related self-efficacy, self-care activities, and quality of life, were collected via a Web-based survey. Details to access this survey were emailed to patients.

4b-ii) Report how institutional affiliations are displayed

5) CONSORT: Describe the interventions for each group with sufficient details to allow replication, including how and when they were actually administered

5-i) Mention names, credential, affiliations of the developers, sponsors, and owners

Patients in both groups were asked to continue with their usual care, which included a visit to their primary diabetes health care practitioner every 3 months. Additionally, patients allocated to the intervention arm were given instructions to download the smartphone application named "Glucose Buddy". Glucose Buddy is a freely available diabetes self-management iPhone application that allows users to manually enter blood glucose levels, insulin dosages, other medications, diet (food item in grams), and physical activities (minutes) [16,23]. Users can also view their data on a customizable graph and export this information via email (Figures 2 and 3). Glucose Buddy was developed by SkyHealth LLC and was first made available on iTunes (Apple online store) in October 2008. Glucose Buddy has been reported to be the most downloaded diabetes management software on iOS, with downloads in excess of 100,000. There was no minimum amount of logging required and intervention patients were able to utilize the accompanying Glucose Buddy website to log diabetes parameters at their discretion.

5-ii) Describe the history/development process

The Glucose Buddy application was developed by a commercial company. We did not conduct usability testing on the application. We used the application because it was the most downloaded (thus most popular) diabetes application at the time of the study.

5-iii) Revisions and updating

There were no revisions or changes to the technology during the intervention.

5-iv) Quality assurance methods

Glucose Buddy (company) gave us back end access to their database so we could source the patients entered information from the application directly. Thus the quality of information was controlled.

5-v) Ensure replicability by publishing the source code, and/or providing screenshots/screen-capture video, and/or providing flowcharts of the algorithms used

Glucose Buddy is a free application on iTunes and they have a website with screenshots of their app.

Glucose Buddy was developed by SkyHealth LLC and was first made available on iTunes (Apple online store) in October 2008. Glucose Buddy has been reported to be the most downloaded diabetes management software on iOS, with downloads in excess of 100,000. There was no minimum amount of logging required and intervention patients were able to utilize the accompanying Glucose Buddy website to log diabetes parameters at their discretion.

5-vi) Digital preservation

Screenshots of the application have been provided to JMIR.

5-vii) Access

Patients in both groups were asked to continue with their usual care, which included a visit to their primary diabetes health care practitioner every 3 months. Additionally, patients allocated to the intervention arm were given instructions to download the smartphone application named "Glucose Buddy". Glucose Buddy is a freely available diabetes self-management iPhone application that allows users to manually enter blood glucose levels, insulin dosages, other medications, diet (food item in grams), and physical activities (minutes) [16,23]. Users can also view their data on a customizable graph and export this information via email (Figures 2 and 3). Glucose Buddy was developed by SkyHealth LLC and was first made available on iTunes (Apple online store) in October 2008. Glucose Buddy has been reported to be the most downloaded diabetes management software on iOS, with downloads in excess of 100,000. There was no minimum amount of logging required and intervention patients were able to utilize the accompanying Glucose Buddy website to log diabetes parameters at their discretion.

5-viii) Mode of delivery, features/functionalities/components of the intervention and comparator, and the theoretical framework

Patients in both groups were asked to continue with their usual care, which included a visit to their primary diabetes health care practitioner every 3 months. Additionally, patients allocated to the intervention arm were given instructions to download the smartphone application named "Glucose Buddy". Glucose Buddy is a freely available diabetes self-management iPhone application that allows users to manually enter blood glucose levels, insulin dosages, other medications, diet (food item in grams), and physical activities (minutes) [16,23]. Users can also view their data on a customizable graph and export this information via email (Figures 2 and 3). Glucose Buddy was developed by SkyHealth LLC and was first made available on iTunes (Apple online store) in October 2008. Glucose Buddy has been reported to be the most downloaded diabetes management software on iOS, with downloads in excess of 100,000.

5-ix) Describe use parameters

There was no minimum amount of logging required and intervention patients were able to utilize the accompanying Glucose Buddy website to log diabetes parameters at their discretion.

5-x) Clarify the level of human involvement

The information logged in the Glucose Buddy application was reviewed by a CDE via a Web interface on a weekly basis. All patients in the intervention arm were sent a minimum of 1 personalized text-message communication per week for the first 6 months of the study. At the 6-month timeframe, all text-message communication ceased.

5-xi) Report any prompts/reminders used

Intervention patients' engagement with the Glucose Buddy application, in terms of the number of logs and text messages communicated between patients and the CDE, is outlined in Table 3. Over the 6-month intervention period, the CDE sent in total 1714 text messages, which equates to approximately 2 text messages per patient per week. Patients sent in total 559 text messages to the CDE over the 6-month period. The first month of the study was used for the CDE and the intervention group patients to establish a relationship—they never met in person. Thus, the text messages sent to patients (mean 9.75, SD 1.96) in the first month and those received by the CDE (mean 6.47, SD 3.92) are higher than the average of the other five months.

5-xii) Describe any co-interventions (incl. training/support)

There was no training or support provided in the use of the technology.

6a) CONSORT: Completely defined pre-specified primary and secondary outcome measures, including how and when they were assessed

All measures were collected at baseline and every three months over the nine-month study period for both groups (making 4 time points in total). The primary outcome measure was change in glycemic control assessed by HbA1c, which was collected by a pathology lab at the request of the patients' general practitioner or endocrinologist as per usual care (every 3 months) and then forwarded to the research team. The secondary outcome measures, being diabetes-related self-efficacy, self-care activities, and quality of life, were collected via a Web-based survey. Details to access this survey were emailed to patients.

6a-i) Online questionnaires: describe if they were validated for online use and apply CHERRIES items to describe how the questionnaires were designed/deployed

All measures were collected at baseline and every three months over the nine-month study period for both groups (making 4 time points in total). The primary outcome measure was change in glycemic control assessed by HbA1c, which was collected by a pathology lab at the request of the patients' general practitioner or endocrinologist as per usual care (every 3 months) and then forwarded to the research team. The secondary outcome measures, being diabetes-related self-efficacy, self-care activities, and quality of life, were collected via a Web-based survey. Details to access this survey were emailed to patients.

6a-ii) Describe whether and how "use" (including intensity of use/dosage) was defined/measured/monitored

Intervention patients' engagement with the Glucose Buddy application, in terms of the number of logs and text messages communicated between patients and the CDE, is outlined in Table 3. Over the 6-month intervention period, the CDE sent in total 1714 text messages, which equates to approximately 2 text messages per patient per week. Patients sent in total 559 text messages to the CDE over the 6-month period. The first month of the study was used for the CDE and the intervention group patients to establish a relationship—they never met in person. Thus, the text messages sent to patients (mean 9.75, SD 1.96) in the first month and those received by the CDE (mean 6.47, SD 3.92) are higher than the average of the other five months. Using the Glucose Buddy application, patients logged 24,720 diabetes parameters in total: 54.00% (13,349/24,720) of the logs related to blood glucose levels, 33.00% (8158/24,720) to insulin, 12.00% (2966/24,720) to diet, and 1.00% (247/24,720) to exercise. Linear regression analysis revealed no significant relationship between level of engagement and change in HbA1c in the intervention, as measured by text messages sent to the patients, text messages received by the CDE, and the number of logs entered in the Glucose Buddy application.

6a-iii) Describe whether, how, and when qualitative feedback from participants was obtained

Qualitative feedback was received through the text messages sent from patients to the CDE.

6b) CONSORT: Any changes to trial outcomes after the trial commenced, with reasons

There were no changes to the trial outcomes after the trial commenced.

7a) CONSORT: How sample size was determined

7a-i) Describe whether and how expected attrition was taken into account when calculating the sample size

The sample size was calculated on the expected difference in mean (1.5%) in the primary outcome variable (HbA1c) and the logistically maximum available sample size was 36 patients per group based on part-time work status of the CDE. We allowed for a dropout of 11% (4 per group), consistent with dropout rates reported in recent reviews of similar studies [9,10], and variation in baseline (HbA1c ~1.80) similar to previous studies [28]. Based on these parameters and using an alpha of .05 and 90% power, the estimated sample size was 68 in total and subsequently increased to 72 in line with the maximum caseload of the CDE [29].

7b) CONSORT: When applicable, explanation of any interim analyses and stopping guidelines

There was no interim analyses.

8a) CONSORT: Method used to generate the random allocation sequence

After confirming eligibility (via phone call) and obtaining written informed consent (via email) from the patient and their primary diabetes health care practitioner (general practitioner or endocrinologist), the study coordinator randomized patients using a freely available online randomization program. A permuted block randomization design method was used during the 3-month rolling recruitment to ensure roughly equal numbers of patients were allocated to each comparison group [22]. There was no face-to-face contact between the patients and research team at any point in the study, which allowed participants to live anywhere in Australia.

8b) CONSORT: Type of randomisation; details of any restriction (such as blocking and block size)

After confirming eligibility (via phone call) and obtaining written informed consent (via email) from the patient and their primary diabetes health care practitioner (general practitioner or endocrinologist), the study coordinator randomized patients using a freely available online randomization program. A permuted block randomization design method was used during the 3-month rolling recruitment to ensure roughly equal numbers of patients were allocated to each comparison group [22]. There was no face-to-face contact between the patients and research team at any point in the study, which allowed participants to live anywhere in Australia.

9) CONSORT: Mechanism used to implement the random allocation sequence (such as sequentially numbered containers), describing any steps taken to conceal the sequence until interventions were assigned

After confirming eligibility (via phone call) and obtaining written informed consent (via email) from the patient and their primary diabetes health care practitioner (general practitioner or endocrinologist), the study coordinator randomized patients using a freely available online randomization program. A permuted block randomization design method was used during the 3-month rolling recruitment to ensure roughly equal numbers of patients were allocated to each comparison group [22]. There was no face-to-face contact between the patients and research team at any point in the study, which allowed participants to live anywhere in Australia.

10) CONSORT: Who generated the random allocation sequence, who enrolled participants, and who assigned participants to interventions

After confirming eligibility (via phone call) and obtaining written informed consent (via email) from the patient and their primary diabetes health care practitioner (general practitioner or endocrinologist), the study coordinator randomized patients using a freely available online randomization program. A permuted block randomization design method was used during the 3-month rolling recruitment to ensure roughly equal numbers of patients were allocated to each comparison group [22]. There was no face-to-face contact between the patients and research team at any point in the study, which allowed participants to live anywhere in Australia.

11a) CONSORT: Blinding - If done, who was blinded after assignment to interventions (for example, participants, care providers, those assessing outcomes) and how

11a-i) Specify who was blinded, and who wasn't

There was no blinding of participants.

11a-ii) Discuss e.g., whether participants knew which intervention was the "intervention of interest" and which one was the "comparator"

Participants were told about the two groups - it was not possible to blind them, as one group got access to the smartphone app and the other group did not.

11b) CONSORT: If relevant, description of the similarity of interventions

Patients in both groups were asked to continue with their usual care, which included a visit to their primary diabetes health care practitioner every 3 months. Additionally, patients allocated to the intervention arm were given instructions to download the smartphone application named "Glucose Buddy". Glucose Buddy is a freely available diabetes self-management iPhone application that allows users to manually enter blood glucose levels, insulin dosages, other medications, diet (food item in grams), and physical activities (minutes) [16,23]. Users can also view their data on a customizable graph and export this information via email (Figures 2 and 3). Glucose Buddy was developed by SkyHealth LLC and was first made available on iTunes (Apple online store) in October 2008. Glucose Buddy has been reported to be the most downloaded diabetes management software on iOS, with downloads in excess of 100,000. There was no minimum amount of logging required and intervention patients were able to utilize the accompanying Glucose Buddy website to log diabetes parameters at their discretion.

12a) CONSORT: Statistical methods used to compare groups for primary and secondary outcomes

Demographic characteristics of participants and baseline data for all measures were compared between both study groups to detect differences at baseline using a series of independent sample t tests and chi-square tests. Logistic regression analyses were conducted to evaluate whether participant characteristics (age [years], duration of diabetes [months/years], gender, insulin pump use [Y/N], and baseline HbA1c) were related to dropout (completed vs didn't complete all assessments) during the study. This statistical method is common when evaluating the characteristics that may be related to attrition examined as a dichotomous outcome. Primary (HbA1c) and secondary outcomes (diabetes-related self-efficacy, quality of life, and self-care) measures were analyzed using linear mixed effects models for repeated measures. Linear mixed model analysis allows for inclusion of cases with missing data, without replacement of missing values, and therefore includes all randomized patients. The linear mixed model analysis used group and the covariates of age (years), gender, and diabetes duration (years) as fixed effects. The "Type III Wald test" was used to test overall statistical significance of the effects. Linear regression analysis was conducted to analyze whether engagement in the study by the intervention group was predictive of change in HbA1c; it allows the assessment of whether patients that engaged more in the intervention in terms of text-message communications and logging parameters in the Glucose Buddy application had a greater change in HbA1c. Statistical significance was defined as $P<.05$ for all analysis and conducted using SPSS for Windows (Version 18.0).

12a-i) Imputation techniques to deal with attrition / missing values

Demographic characteristics of participants and baseline data for all measures were compared between both study groups to detect differences at baseline using a series of independent sample t tests and chi-square tests. Logistic regression analyses were conducted to evaluate whether participant characteristics (age [years], duration of diabetes [months/years], gender, insulin pump use [Y/N], and baseline HbA1c) were related to dropout (completed vs didn't complete all assessments) during the study. This statistical method is common when evaluating the characteristics that may be related to attrition examined as a dichotomous outcome. Primary (HbA1c) and secondary outcomes (diabetes-related self-efficacy, quality of life, and self-care) measures were analyzed using linear mixed effects models for repeated measures. "Linear mixed model analysis allows for inclusion of cases with missing data, without replacement of missing values, and therefore includes all randomized patients. "

12b) CONSORT: Methods for additional analyses, such as subgroup analyses and adjusted analyses

Demographic characteristics of participants and baseline data for all measures were compared between both study groups to detect differences at baseline using a series of independent sample t tests and chi-square tests. Logistic regression analyses were conducted to evaluate whether participant characteristics (age [years], duration of diabetes [months/years], gender, insulin pump use [Y/N], and baseline HbA1c) were related to dropout (completed vs didn't complete all assessments) during the study. This statistical method is common when evaluating the characteristics that may be related to attrition examined as a dichotomous outcome. Primary (HbA1c) and secondary outcomes (diabetes-related self-efficacy, quality of life, and self-care) measures were analyzed using linear mixed effects models for repeated measures. Linear mixed model analysis allows for inclusion of cases with missing data, without replacement of missing values, and therefore includes all randomized patients. The linear mixed model analysis used group and the covariates of age (years), gender, and diabetes duration (years) as fixed effects. The "Type III Wald test" was used to test overall statistical significance of the effects. Linear regression analysis was conducted to analyze whether engagement in the study by the intervention group was predictive of change in HbA1c; it allows the assessment of whether patients that engaged more in the intervention in terms of text-message communications and logging parameters in the Glucose Buddy application had a greater change in HbA1c. Statistical significance was defined as $P<.05$ for all analysis and conducted using SPSS for Windows (Version 18.0).

RESULTS

13a) CONSORT: For each group, the numbers of participants who were randomly assigned, received intended treatment, and were analysed for the primary outcome

See Figure 1 and Table 1 for number of participants randomly assigned, receiving intended treatment.

Linear mixed model analysis allows for inclusion of cases with missing data, without replacement of missing values, and therefore includes all randomized patients.

13b) CONSORT: For each group, losses and exclusions after randomisation, together with reasons

See Figure 1: Participant Flow: note: Reason for subject "lost to follow-up" could not be determined as patients could not be recontacted."

13b-i) Attrition diagram

see Figure 1 for attrition and Table 3 for engagement over time.

14a) CONSORT: Dates defining the periods of recruitment and follow-up

Original data was collected November 2010 to November 2011 and analysed in 2012.

14a-i) Indicate if critical "secular events" fell into the study period

There were no critical secular event during the study period.

14b) CONSORT: Why the trial ended or was stopped (early)

N/A

15) CONSORT: A table showing baseline demographic and clinical characteristics for each group

see Table 1.

15-i) Report demographics associated with digital divide issues

see Table 1.

16a) CONSORT: For each group, number of participants (denominator) included in each analysis and whether the analysis was by original assigned groups

16-i) Report multiple "denominators" and provide definitions

see Figure 1 and Table 1.

Linear mixed model analysis allows for inclusion of cases with missing data, without replacement of missing values, and therefore includes all randomized patients.

16-ii) Primary analysis should be intent-to-treat

Linear mixed model analysis allows for inclusion of cases with missing data, without replacement of missing values, and therefore includes all randomized patients.

17a) CONSORT: For each primary and secondary outcome, results for each group, and the estimated effect size and its precision (such as 95% confidence interval)

The sample size was calculated on the expected difference in mean (1.5%) in the primary outcome variable (HbA1c) and the logistically maximum available sample size was 36 patients per group based on part-time work status of the CDE. We allowed for a dropout of 11% (4 per group), consistent with dropout rates reported in recent reviews of similar studies [9,10], and variation in baseline (HbA1c ~1.80) similar to previous studies [28]. Based on these parameters and using an alpha of .05 and 90% power, the estimated sample size was 68 in total and subsequently increased to 72 in line with the maximum caseload of the CDE [29].

17a-i) Presentation of process outcomes such as metrics of use and intensity of use

see Table 3 and 4

17b) CONSORT: For binary outcomes, presentation of both absolute and relative effect sizes is recommended

N/A

18) CONSORT: Results of any other analyses performed, including subgroup analyses and adjusted analyses, distinguishing pre-specified from exploratory

In total, 197 adults with type 1 diabetes registered their interest online or via phone call to the research team and were assessed for eligibility (Figure 1), with 125 excluded for not meeting the inclusion criteria. Seventy-two individuals were randomized to the two groups. Linear mixed model analysis allows for inclusion of cases with missing data, without replacement or imputation of missing values. Therefore, this analysis approach includes all available data of randomized patients at each time point as indicated in Figure 1. Table 1 provides an outline of the participant's characteristics. Mean age of patients was 35.20 years (SD 10.43), 39% (28/72) were male, and patients had been diagnosed with type 1 diabetes for a mean of 18.94 years (SD 9.66). In total, 37.5% (27/72) of patients were using an insulin pump, with no significant difference between groups, $\chi^2 1=0.59$, P=.81. The intervention group had a significantly higher (P=.02) baseline HbA1c (mean 9.08, SD 1.18) than the control group (mean 8.47, SD 0.86) and reported a healthier diet (mean 3.56, SD 1.70 healthy days per week for the intervention group versus mean 2.60, SD 1.98 days for the control group, P=.03). There were significantly (P=.02) more females (75%, 27/36) in the control group. No other baseline differences were observed between groups. Dropout was 26% (11 males, 8 females, 19/72) with logistic regression analysis revealing no significant difference in age, gender, diabetes duration, insulin pump use, and baseline HbA1c among those that completed the study and those that were lost to follow up.

18-i) Subgroup analysis of comparing only users

Intervention patients' engagement with the Glucose Buddy application, in terms of the number of logs and text messages communicated between patients and the CDE, is outlined in Table 3. Over the 6-month intervention period, the CDE sent in total 1714 text messages, which equates to approximately 2 text messages per patient per week. Patients sent in total 559 text messages to the CDE over the 6-month period. The first month of the study was used for the CDE and the intervention group patients to establish a relationship—they never met in person. Thus, the text messages sent to patients (mean 9.75, SD 1.96) in the first month and those received by the CDE (mean 6.47, SD 3.92) are higher than the average of the other five months. Using the Glucose Buddy application, patients logged 24,720 diabetes parameters in total: 54.00% (13,349/24,720) of the logs related to blood glucose levels, 33.00% (8158/24,720) to insulin, 12.00% (2966/24,720) to diet, and 1.00% (247/24,720) to exercise. Linear regression analysis revealed no significant relationship between level of engagement and change in HbA1c in the intervention, as measured by text messages sent to the patients, text messages received by the CDE, and the number of logs entered in the Glucose Buddy application.

19) CONSORT: All important harms or unintended effects in each group

There were no unintended effects to the participants.

19-i) Include privacy breaches, technical problems

There were no privacy breaches or technical problems.

19-ii) Include qualitative feedback from participants or observations from staff/researchers

This is outside the scope of the study.

DISCUSSION

20) CONSORT: Trial limitations, addressing sources of potential bias, imprecision, multiplicity of analyses

20-i) Typical limitations in ehealth trials

There are limitations to our study that should be noted. First, this study was a randomized controlled trial with a small sample conducted over a short duration. Due to the dropout of patients, the study may not have been powered sufficiently to detect differences between groups for the secondary outcome measures. Second, there were differences in glycemic control and gender between groups at baseline. Third, although patients in the control group were instructed not to use any mobile applications to self-manage their diabetes during the study period, it is possible they did.

21) CONSORT: Generalisability (external validity, applicability) of the trial findings

21-i) Generalizability to other populations

Despite these limitations, we did find that integrating a smartphone application into secondary care was effective in improving glycemic control in patients with type 1 diabetes. Our findings can be applied to adults with poorly controlled type 1 diabetes that own a smartphone, though larger studies over a longer duration need to be conducted to validate our findings.

21-ii) Discuss if there were elements in the RCT that would be different in a routine application setting

N/A

22) CONSORT: Interpretation consistent with results, balancing benefits and harms, and considering other relevant evidence

22-i) Restate study questions and summarize the answers suggested by the data, starting with primary outcomes and process outcomes (use)

In adjunct with usual care, use of the Glucose Buddy application combined with weekly text-message feedback from a CDE led to a significant decrease in HbA1c in comparison to a control group receiving only usual care. While regression to the mean cannot be ruled out, these results suggest that the intervention was effective. Improvements in HbA1c of this magnitude in type 1 diabetes patients have been found previously in a mobile phone study [2] but are rare [17,19,30,31]. All patients in our study had poorly controlled diabetes at baseline; however, the intervention group had a significantly higher HbA1c at baseline (mean 9.08%, SD 1.18 vs 8.47%, SD 0.86) and thus had a greater potential to improve their glycemic control. However, a meta-analysis of mobile intervention studies on diabetes glycemic control demonstrated only a 0.3% improvement for type 1 patients [9]; this demonstrates the success of the current intervention (a decrease of 1.1% in the intervention group) despite the baseline differences observed between groups.

22-ii) Highlight unanswered new questions, suggest future research

Our findings can be applied to adults with poorly controlled type 1 diabetes that own a smartphone, though larger studies over a longer duration need to be conducted to validate our findings.

Other information

23) CONSORT: Registration number and name of trial registry

Trail Registration: Australian New Zealand Clinical Trials Registry: ACTRN12612000132842

24) CONSORT: Where the full trial protocol can be accessed, if available

https://www.anzctr.org.au/Trial/Registration/TrialReview.aspx?ACTRN=12612000132842

25) CONSORT: Sources of funding and other support (such as supply of drugs), role of funders

This study was funded by Central Queensland University, Australia. The authors thank Certified Diabetes Educator Veronica Mills (Queensland Health) and SkyHealth, the developers of Glucose Buddy application and website.

C Vandelanotte is supported by a National Health and Medical Research Council of Australia (#519778) and National Heart Foundation of Australia (#PH 07B 3303) postdoctoral research fellowship. M Kirwan is supported by a Queensland Government, Department of Tourism, Regional Development and Industry, SmartFutures PhD Scholarship.

X26-i) Comment on ethics committee approval

This study was approved by Central Queensland University Human Research Ethics Board.

x26-ii) Outline informed consent procedures

After confirming eligibility (via phone call) and obtaining written informed consent (via email) from the patient and their primary diabetes health care practitioner (general practitioner or endocrinologist), the study coordinator randomized patients using a freely available online randomization program. A permuted block randomization design method was used during the 3-month rolling recruitment to ensure roughly equal numbers of patients were allocated to each comparison group [22]. There was no face-to-face contact between the patients and research team at any point in the study, which allowed participants to live anywhere in Australia.

X26-iii) Safety and security procedures

N/A

X27-i) State the relation of the study team towards the system being evaluated

The authors declare that there is no duality of interest associated with this manuscript.
